# circCRKL, a circRNA derived from *CRKL,* regulates BCR-ABL via sponging miR-877-5p to promote chronic myeloid leukemia cell proliferation

**DOI:** 10.1186/s12967-022-03586-2

**Published:** 2022-09-04

**Authors:** Jianming Wang, Yang Liang, Yuefeng Qin, Guoyun Jiang, Yuhang Peng, Wenli Feng

**Affiliations:** Department of Clinical Hematology, Key Laboratory of Laboratory Medical Diagnostics Designated by the Ministry of Education, Chongqing Medical University, Chongqing, 400016 People’s Republic of China

**Keywords:** Chronic myeloid leukemia, circCRKL, Proliferation, BCR-ABL, ceRNA

## Abstract

**Background:**

The BCR-ABL fusion protein is the key factor that results in the occurrence of chronic myeloid leukemia (CML). Imatinib (IM) is a targeted inhibitor of BCR-ABL to achieve complete remission. However, remission failure occurs due to acquired resistance caused by secondary BCR-ABL mutations, underlining the need for novel BCR-ABL-targeting strategies. Circular RNAs (circRNAs) derived from tumor-related genes have been revealed as possible therapeutic targets for relevant cancers in recent investigations. In CML, the roles of this kind of circRNA are yet obscure.

**Methods:**

Firstly, RT-qPCR was used for determining circCRKL expression level in cell lines and clinical samples, RNase R and Actinomycin D were employed to verify the stability of circCRKL. Then shRNAs were designed to specifically knockdown circCRKL. The function of circCRKL in vitro was investigated using CCK-8, colony formation assay, and flow cytometry, while a CML mouse model was constructed to explore the function in vivo. Finally, a dual-luciferase reporter assay, RNA pull-down, RNA immunoprecipitation, and rescue experiments were conducted to investigate the mechanism of circCRKL functioning.

**Results:**

Here, we determined circCRKL, which derives from CML-relevant gene *CRKL*, is over-expressed in BCR-ABL^+^ cells. Then we noticed knocking down circCRKL using shRNA lentivirus dampens the proliferation of BCR-ABL^+^ cells both in vitro and in vivo, and augments susceptibility of resistant cells to IM. Intriguingly, we observed that circCRKL has a considerable impact on the expression level of BCR-ABL. Mechanistically, circCRKL could behave like a decoy for miR-877-5p to enhance the BCR-ABL level, allowing BCR-ABL^+^ cells to maintain viability.

**Conclusions:**

Overall, the current study uncovers that circCRKL is specifically expressed and regulates BCR-ABL expression level via decoying miR-877-5p in BCR-ABL^+^ cells, highlighting that targeting circCRKL along with imatinib treatment could be utilized as a potential therapeutic strategy for CML patients.

**Supplementary Information:**

The online version contains supplementary material available at 10.1186/s12967-022-03586-2.

## Background

Chronic myeloid leukemia (CML) was identified as a neoplastic disease of hematopoietic stem cells, arising from a fusion event termed *BCR-ABL* [1]. The oncogenic fusion protein BCR-ABL with aberrant tyrosine kinase function is encoded by the *BCR-ABL* [2], leading to constitutively activation of various substrates including CRKL, PI3K/AKT, and MAPK signaling pathways, resulting in malignant cell proliferation [3–5]. Tyrosine kinase inhibitor (TKI) imatinib mesylate (IM), which has the benefit of significantly enhancing patient prognoses, has been used extensively in the clinical management of CML individuals [6]. However, individuals receiving long-term IM treatment risk developing resistance since BCR-ABL (the root cause of CML) cannot be entirely eradicated. Imatinib resistant CML patients had a significantly increased risk of relapse after discontinuation [7, 8]. The pathogenesis of CML is well understood, however, the underlying molecular mechanisms during disease development and TKI resistance are required to be clarified, thus providing novel insights for diagnosis and therapy in CML.

In the last few decades, the underlying mechanisms of CML progression and TKI resistance predominantly focused on messenger RNAs (mRNAs) that encode specific proteins [9, 10] or long non-coding RNAs (lncRNAs) [11, 12] or micro RNAs (miRNAs). miR-185, for example, has been uncovered to function as a repressor of PAK6, mediating Imatinib resistance in TKIs resistant leukemic cells, indicating that miR-185 might be a novel target for treating TKIs resistant CML patients [13]. As a result of the rapid advancement of high-throughput sequencing, circular RNAs (circRNAs) have come into view. As a unique kind of regulating non-coding RNAs [14], circRNA lacks the classic cap and poly-A tail structure retained by linear RNAs [15]. A stable circular form renders circRNA more suitable as a molecular target [16]. Accumulating evidence elucidated that circRNAs were extensively engaged in regulating the development of many malignancies, including liver cancer [17], lung cancer [18], and leukemia [19, 20]. Diverse functions of circRNAs are rested with different subcellular localization, including sponging miRNA, acting as a scaffold to decoy RNA binding proteins (RBPs) and translated peptides in the cytoplasm, while mediating transcription of parental genes in the nucleus [14, 21–23].

Amounts of evidence illustrated circRNAs derived from tumor-related parental genes can regulate the onset and progression of malignancies. For example, circMYBL2, produced from *MYBL2*, which was reported to be linked to leukemia, could promote the advancement of *FLT3*-ITD-mutant acute myeloid leukemia [20]. In addition, by stimulating the Wnt signal pathway, circ β-catenin, which was generated from *CTNNB1*, translated a novel β-catenin isoform and accelerated hepatocellular carcinoma cell growth [24]. In CML, the roles of circRNA are yet obscure. It was suggested that fusion-circRNA (f-circRNA) derived from *BCR-ABL* participated in regulating the progression of CML [25, 26]. However, few pieces of evidence uncovered that the roles of circRNAs originated from other known genes in *BCR-ABL* cascades, such as *CRKL*.

*CRKL* also termed *CRK*-like gene, produces an oncogenic protein CRKL that has been pinpointed as a predominant element of tyrosine phosphorylation [27]. It has been suggested that CRKL complexes directly with BCR-ABL via the SH3 domain, triggering signaling cascades such as PI3K-AKT pathway that contribute to leukemic transformation [28]. Besides, CRKL lacking the SH2 domain reduced c-MYC expression level in imatinib-resistant cells harboring BCR-ABL T315I mutation [29]. Recent studies showed that hsa_circ_0001206, produced by the second exon of *CRKL* pre-mRNA, was a tumor suppressor and restrained cell growth by regulating KLF5 through mediating miR-141 in prostate cancer [30]. Besides, several lines of evidence suggested that circCRKL blocked AML progression through targeting miR-196-5p [31].

In this work, we discovered that circCRKL, a circRNA that originated from the second exon of *CRKL*, is significantly highly expressed and accelerates the BCR-ABL^+^ cells proliferation including imatinib-resistant cell line K562/G01. Our data further confirmed that circCRKL modulates BCR-ABL via decoying miR-877-5p to hasten cell proliferation. Therefore, our findings suggested that circCRKL impels CML progression by acting an oncogenic role, and targeting circCRKL might represent a novel therapeutic option for CML patients.

## Materials and methods

### Cell culture and management

Human chronic myeloid leukemia cell lines (K562, K562/G01, and KCL22), acute monocytic leukemia cell line (THP-1), human lymphoblastic cell line (TK6), and 293 T were all maintained in Roswell Park Memorial Institut-1640 (RPMI-1640, Gibco, USA) or Dulbecco's modified eagle medium (DMEM, Gibco, USA) with 10% fetal bovine serum (FBS, Gibco, USA) respectively. All cells were cultivated at 37 °C in a suitable atmosphere containing 5% CO_2_. The Chinese Academy of Science's Cell Bank provided all of the cell lines utilized in this work.

The bone marrow samples of newly diagnosed CML patients and normal donors were obtained from the Department of Hematology, the Second Affiliated Hospital of Chongqing Medical University. The information was illustrated in Additional file 3: Table S1. The Human bone marrow mononuclear cells (BMMCs) separation Kit (TBD, China) was used to isolate BMMCs from the samples. Before the trial began, all patients signed informed permission forms. The ethics committee of Chongqing Medical University gave its approval to this investigation.

### RNA extraction and RT-qPCR

TRizol reagent (Accurate, China) was used to extract total RNA from patient samples and cultured cells. To reverse transcribe circRNA and messenger RNA (mRNA) into complementary DNA (cDNA), a reverse transcription kit (Accurate, China) was employed. The stem-loop primer was used to reverse transcribe miR-877-5p. The quantitative PCR was carried out through SYBR Green Premix Pro Taq HS qPCR Kit (Accurate, China) following the product’s instructions. *Actin* or *U6* was used as RT-qPCR internal parameters. The 2^−ΔΔCT^ method was utilized to examine the relative expression of RNA. All specific primers sequences used in this investigation were listed in Additional file 3: Table S2.

### RNase R resistance and Actinomycin D analysis of circCRKL

The 1 μg of total RNA extraction of K562, and K562/G01 cells were digested at 37 °C for 10 min after adding 2U/mg of RNase R (Geneseed, China), then the mixture was hatched at 70 °C for another 10 min to inactive the RNase R. Subsequently, the relative RNA enrichment was detected through RT-qPCR.

The 5 μg/mL actinomycin D was mixed with K562 and K562/G01 cells and reacted for 4, 8, and 12 h respectively, the total RNA was then extracted. The relative RNA expression was then identified using RT-qPCR.

### Fluorescence in situ hybridization (FISH)

The specific circCRKL probe was labeled with cy3 and synthesized by RiboBio (Guangzhou, China). The assay was conducted in accordance with the manufacturer’s orders. In brief, K562 and K562/G01 cells were washed and fixed with 4% paraformaldehyde at room temperature (RT) for 10 min before being permeabilized with 0.5% Triton X-100 at 4 °C for 5 min. Then circCRKL probe was hybridized with cells in a dark moist chamber at 37 °C overnight. To stain the nuclei, 4′,6-diamidino-2-phenylindole (DAPI) was used. A confocal microscope was utilized to acquire photos.

### Infection of lentivirus

For circCRKL down-regulation, small interfering oligos targeting circCRKL (the sequences are illustrated in Additional file 3: Table S3) were designed and constructed into lentivirus (GeneChem, China), which were subsequently infected into BCR-ABL^+^ and THP-1 cells. Stable transfection was established through culturing cells in RPMI-1640 medium supplemented with 2 μg/ml puromycin (Solarbio, China) for 7 days, and maintained with 1 µg/ml of that for another 7 days.

### CCK-8 assay

The cell viability was determined using a CCK-8 kit (TargetMol, China). Cells were seeded at the number of 3000 per well in a 96-well plate and cultured in an incubator for 24 h before receiving treatment of 10 μl CCK-8 reagent for 3 h. Subsequently, the absorbance was assessed at 450 nm. Three duplicates were set in each group.

### Colony formation assay

Cells were seeded at a density of 100 per well into a 96-well plate. After being stilling cultured using RMPI-1640 with 10% FBS for 7 days, photos were taken and the number of colonies was counted. At least three duplicates were set in each group.

### Flow cytometry assay

For cell cycle assay, at least 1 × 10^6^ infected K562 and K562/G01 cells were immobilized through pre-cooled 70% ethanol at 4 °C overnight. After that, each sample was left in a lucifugal chamber at RT for 30 min after receiving 500 μl of propidium iodide (PI) staining reagent. Then cell cycle was detected and analyzed through a flow cytometer (CytoFlex, Beckman coulter).

For cell apoptosis assay, at least 5 × 10^5^ infected K562 and K562/G01 cells were collected and washed, then cells were suspended with 100 μl pre-cooled PBS and treated with 10 μl Annexin V APC and 5 μl DAPI in the dark at RT for 15 min before being determined with a flow cytometer (CytoFlex, Beckman coulter).

### RNA Immunoprecipitation (RIP)

K562/G01 cells were used to conducted RIP assay with the BersinBio™ RNA-Binding Protein Immunoprecipitation Kit (BersinBio, China) in accordance with the manufacturer’s protocols. In a nutshell, at least 2 × 10^7^ cells were harvested, washed, and lysed using 900 μl RIP lysis buffer, then cell lysates were added with 4 μg of anti-AGO2 (Abcam, USA) or the equal amounts of anti-IgG (BersinBio) and incubated at 4 °C overnight. The mixture was subsequently added with 40 μl protein A/G magnetic beads and incubated at 4 °C for 2 h. Finally, the RNAs were extracted and purified using phenol–chloroform and the relative abundance of miR-877-5p and circCRKL was examined through RT-qPCR.

### Western blot analysis

To lyse cells, a radio immunoprecipitation assay (RIPA) lysing solution including phosphatase and protease inhibitor was employed, and total protein was then extracted and quantified through a bicinchoninic acid (BCA) protein extraction assay kit (Beyotime, China). For western blot assay, 40 μg of protein samples were loaded and isolated with 8% sodium dodecyl sulfate–polyacrylamide gel electrophoresis (SDS-PAGE). Subsequently, blocking the polyvinylidene fluoride (PVDF) membranes containing protein blots with 5% nonfat milk powder at RT for 2 h before incubating the primary antibody (1:1000) at 4 °C overnight. After that, the membranes were incubated with relevant secondary antibodies (1:5000) at RT for 90 min. Finally, the Bio-Rad Gel Imaging System was used to visualize and analyze the signals.

### Bioinformatics prediction and dual-luciferase reporter assay

For the prediction of target miRNAs directly cooperating with circCRKL, three online databases including ENCORI (http://starbase.sysu.edu.cn/), circInteractome (https://circinteractome.nia.nih.gov/), and circBank (http://www.circbank.cn/) were employed. Another two databases TargetScan (http://www.targetscan.org/vert_72/), miRwalk (http://mirwalk.umm.uni-heidelberg.de/), and ENCORI were utilized to search the miRNAs interacting with *ABL*. The overlapping target miRNAs were analyzed and visualized using VennDiagram tools.

The wild-type (WT) reporters of circCRKL and *ABL* containing binding sites with miR-877-5p or mutant (MUT) ones were cloned into the pmiR-GLO luciferase vector (Genecreate, China). The vectors along with miR-NC or miR-877-5p mimic (RiboBio, China) were then transfected into 293 T cells. The Dual-luciferase Reporter Assay System (Promega, USA) was employed to evaluate the luciferase activities after culturing for 2 days.

### Biotinylated RNA pulldown assay

The specific probe targeting the back-splicing site of circCRKL and NC oligo probe was coupled by biotin and purchased from RiboBio (Guangzhou, China). The probe sequences were demonstrated in Additional file 3: Table S4. Briefly, approximately 4 × 10^7^ K562/G01 cells were harvested and fixed using 1% formaldehyde, then the cells were lysed, sonicated, and centrifuged at 16,000*g* at 4 °C for 15 min. The biotin-coupled circCRKL probe or NC oligo probe was incubated with the supernatant obtained from lysates at RT for 4 h. After that, streptavidin-coated magnetic beads (MCE, USA) were utilized to pull down the biotin-labeled RNA mixture. Finally, with the elution of the beads, the RNA was obtained using TRizol, and the relative abundance of circCRKL and miR-877-5p was evaluated with RT-qPCR.

### Xenograft leukemogenesis model

Female NOD/SCID mice aged 5 to 6 weeks were irradiated with an X-ray at a dose of 250 cGy before injection. K562/G01 cells were collected and washed with sterile PBS at a concentration of 2.5 × 10^7^/ml after being infected with sh-NC or sh-circCRKL lentivirus. The mice were then split into two groups at random and intravenously injected with 200 μl cells, respectively. The body weights of each mouse were monitored weekly. The number of white blood cells in the peripheral blood was measured three weeks after injection. The bone marrow cells, livers, and spleens were harvested while mice were closely culled at an ethical endpoint. The animal experiments were given approval by the Ethics Committee of Chongqing Medical University.

### Statistical analysis

GraphPad Prism 8 (GraphPad, USA) was used for analyzing data in this research. The student’s t-test was applied for calculating the main effect in two groups and One-Way ANOVA was used for that in three and more. p-values < 0.05 (*), p < 0.01 (**), p < 0.001 (***) and p < 0.0001 (****) were regarded as statistically significant.

## Results

### Characterization of circCRKL in CML cell lines and clinical samples

**Fig. 1:**
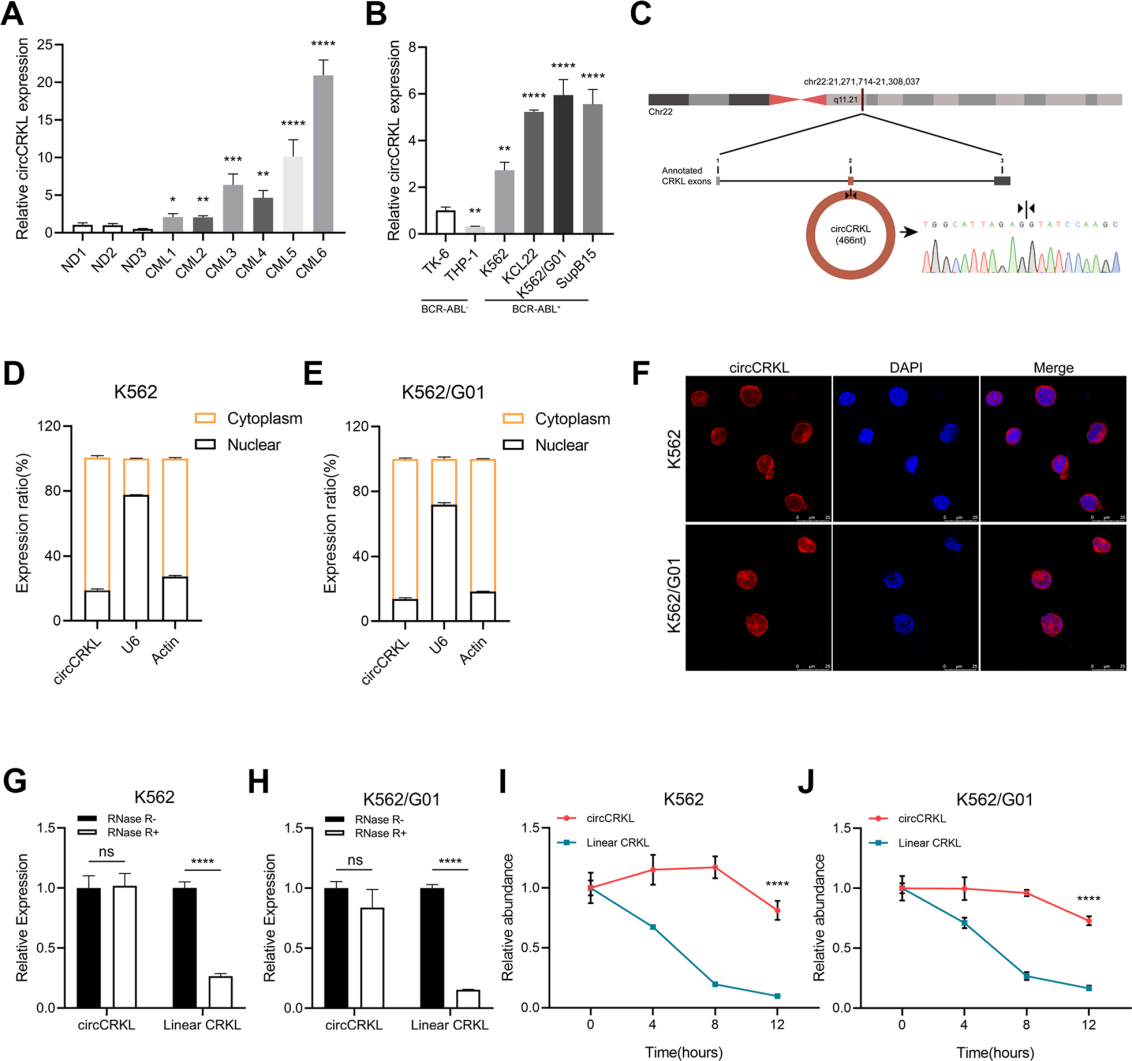
Identifying and characterizing circCRKL in BCR-ABL^+^ cell lines and CML samples. **A** Differential expression of circCRKL between CML and normal donors samples. **B** Relative circCRKL expression in a normal lymphoblastic cell line (TK6) and BCR-ABL^−^ leukemia cell line (THP-1) and BCR-ABL^+^ leukemia cell lines (K562, K562/G01, KCL22, and SupB15). **C** Schematic diagram of circularization of circCRKL, the back-splice site was identified using sanger sequencing. The cytoplasmic distribution of circCRKL in K562 cells (**D**) and K562/G01 cells (**E**). **F** FISH assay demonstrated that circCRKL was predominantly expressed in cytoplasm in K562 and K562/G01 cells (Cy3 was used for labeling circCRKL probe). **G**,** H** After treating K562 and K562/G01 cells with or without RNase R, the relative expression of circCRKL and linear CRKL was measured. **I**,** J** Relative expression of circCRKL and linear CRKL following treatment with actinomycin D in K562 and K562/G01 cells. *p < 0.05, **p < 0.01, ***p < 0.001 and **** < 0.0001

To uncover the hidden role of circCRKL in CML, the circCRKL expression in BMMCs from CML patients (6 cases) and normal donors (3 cases) was detected by RT-qPCR. CircCRKL was substantially expressed in CML patients relative to normal donors, as demonstrated in Fig. 1A. Furthermore, when comparing BCR-ABL^+^ cell lines (K562, K562/G01, KCL22, and SupB15) to BCR-ABL^−^ cell lines (TK6 and THP-1), it was surprising to find that the circCRKL expression was significantly higher in BCR-ABL^+^cell lines (Fig. 1B). The BCR-ABL expression level of cell lines was evaluated by western blot assays (Additional file 1: Figure S1A). These findings suggested that a link may exist between circCRKL and BCR-ABL. Then we conducted a bioinformatics analysis of the *CRKL* transcript. It was found that circCRKL is a 466-nt circRNA and was back spliced by exon 2 of pre-*CRKL*, Sanger sequencing was applied to confirm the cyclization site (Fig. 1C). The results of nuclear and cytoplasm RNA separation assays with RT-qPCR uncovered that circCRKL expression level in the cytoplasm was higher than that in the nucleus in CML cells (Fig. 1D, E), which was further supported by fluorescence in situ hybridization (FISH) assays (Fig. 1F). Subsequently, to determine the circular form of circCRKL, the RNase R analysis was performed and the linear CRKL expression diminished dramatically while circCRKL had no changes, indicating that circCRKL was stable in CML cells (Fig. 1G, H). In addition, the half-life of circCRKL and linear CRKL was determined with the treatment of actinomycin D. It was found that circCRKL had a longer half-life compared with that of linear CRKL (Fig. 1I, J). These findings imply that circCRKL, located in the cytoplasm in CML cells and highly expressed, is a stable circRNA, indicating a potential role in CML.

### circCRKL promotes BCR-ABL^+^ cells proliferation in vitro

**Fig. 2:**
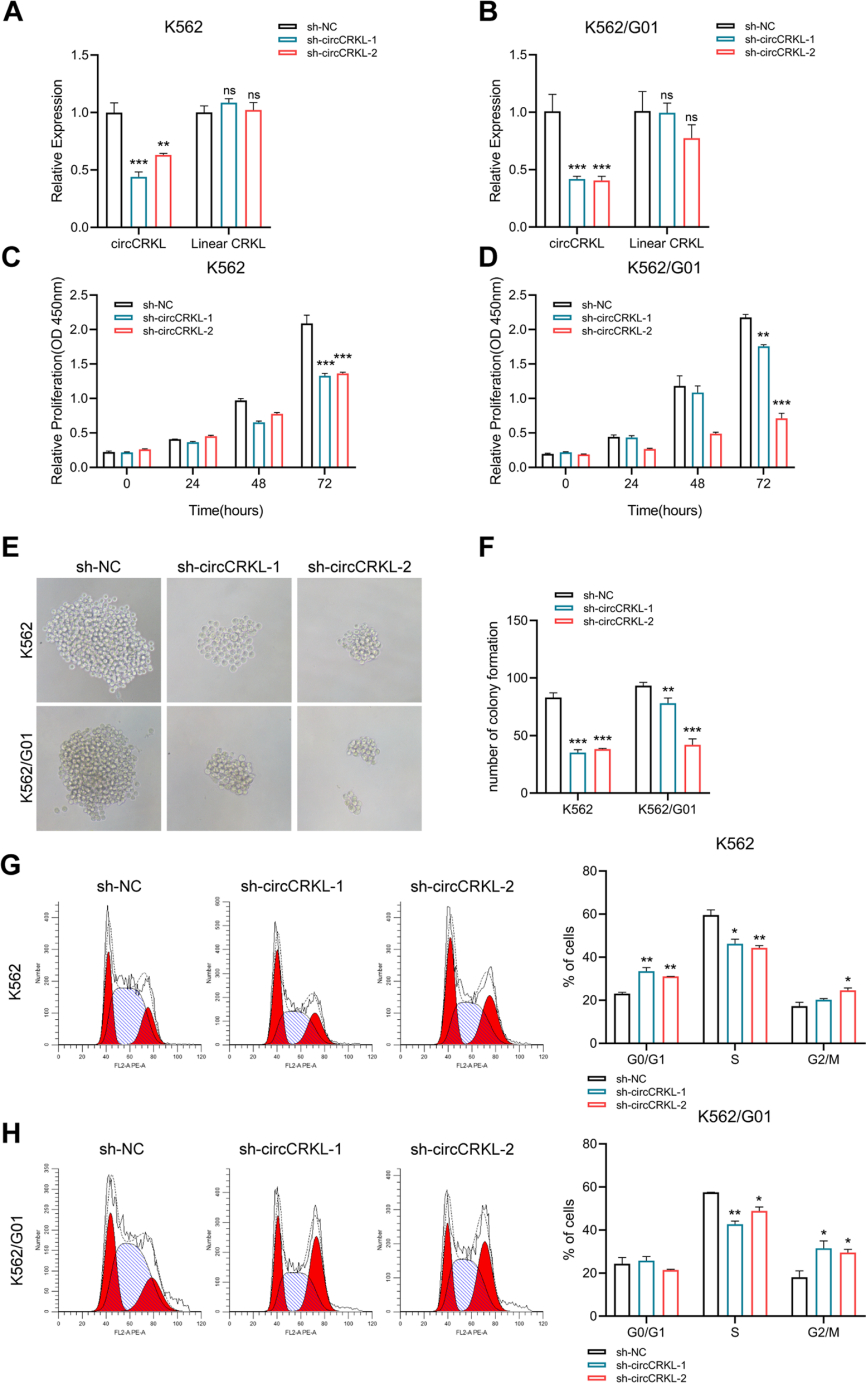
circCRKL promotes CML cells proliferation in vitro. The efficient and specific knockdown of circCRKL in K562 (**A**) and K562/G01 (**B**) cells. **C**,** D** CCK-8 assays were used to investigate the effect of circCRKL knockdown on growth in K562 (**C**) and K562/G01 (**D**) cells. **E**,** F** Effect of circCRKL knockdown on proliferation in K562 and K562/G01 cells was determined by colony formation assays. **G**,** H** Flow cytometry was used to analyze the effect of circCRKL suppression on the cell cycle. *p < 0.05, **p < 0.01, and ***p < 0.001

To further explore the biological involvement of circCRKL in CML, a loss-of-function test was performed. We constructed circCRKL knockdown CML cells using shRNA specifically targeting the back spliced junction site of circCRKL. The expression of circCRKL was markedly restrained in CML cells, whereas the linear CRKL expression was unaffected (Fig. 2A, B). Subsequently, CCK-8 and colony formation assays uncovered that circCRKL suppression drastically reduced CML cell growth (Fig. 2C−F). Flow cytometry assay showed that circCRKL silencing reduced the number of cells in the S phase (Fig. 2G, H). The effect of circCRKL on apoptosis in CML cells was then investigated, and we discovered that the second shRNA of circCRKL silencing engendered cell apoptosis rate increasing (Additional file 1: Figure S1B).

The differential expression of circCRKL in BCR-ABL^+^ and BCR-ABL^−^ cell lines raises the question of whether circCRKL specifically actives in cells expressing BCR-ABL. Hence, the effects of circCRKL silencing on cell viability were investigated in two other BCR-ABL^+^ cell lines (KCL22 and SupB15) as well as BCR-ABL^−^ cell lines (THP-1 and TK-6). The knockdown efficiency of circCRKL was confirmed in these cell lines (Additional file 1: Figure S1C). Next, CCK-8 assays showed circCRKL suppression substantially impaired cell viability of BCR-ABL^+^ cell lines but had no influence on BCR-ABL^−^ cells (Additional file 1: Figure S1D–G). Collectively, these findings imply that circCRKL is specifically required in BCR-ABL^+^ cells, and dampening circCRKL inhibits proliferation.

### circCRKL silencing impaired the oncogenesis of CML cells in vivo

**Fig. 3:**
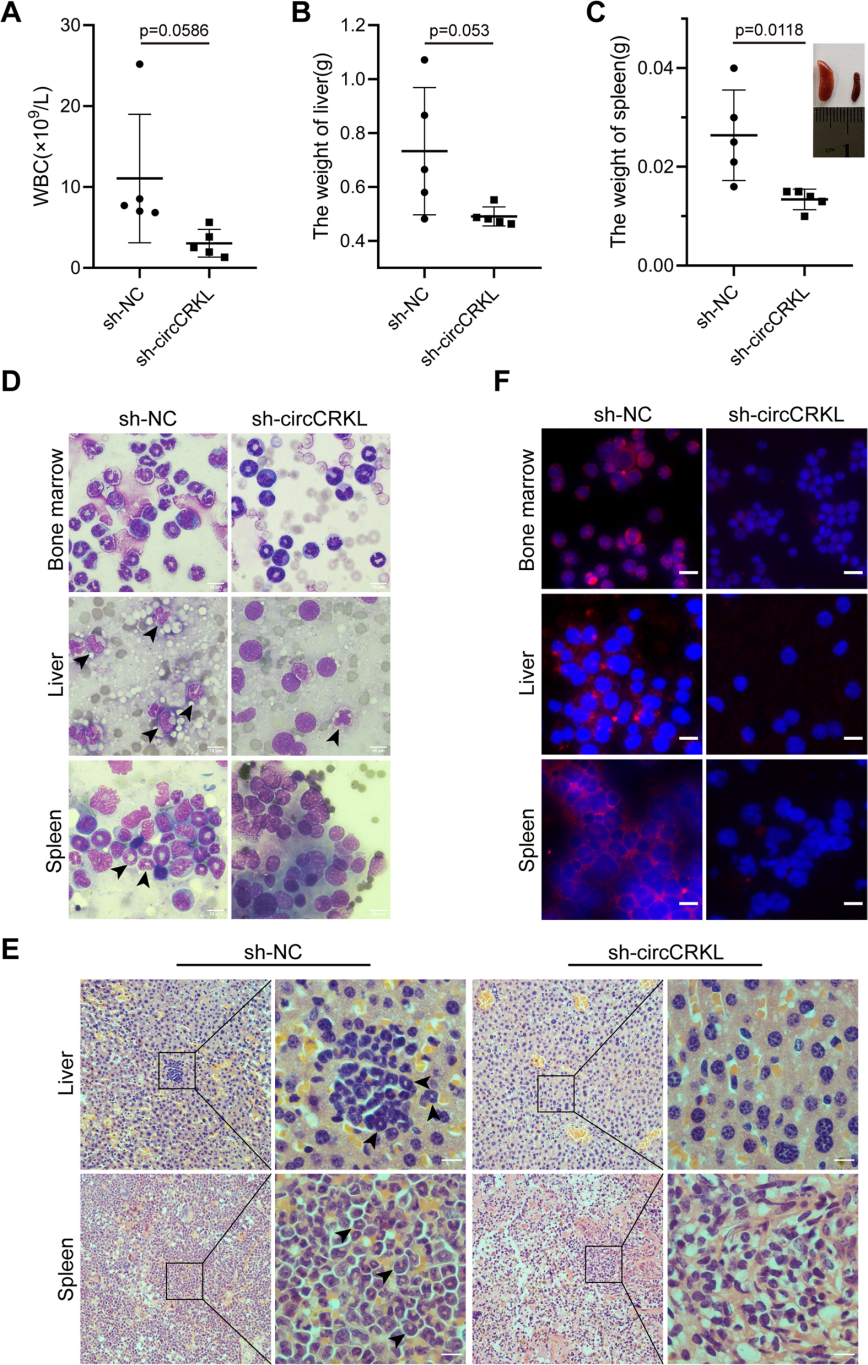
circCRKL enhances CML cells proliferation in vivo. **A** The number of WBCs was calculated. **B**,** C** The weights of liver and spleen were recorded and the images were shown. **D** Wright’s staining was performed to observe the leukemic cells in bone marrow, liver, and spleen, the leukemic cells were indicated by the black arrow. Scale bar, 10 μm. **E** The leukemic infiltration in liver and spleen was observed by H&E staining. **F** BCR-ABL levels in bone marrow, liver, and spleen cells were determined with immunofluorescence. Scale bar, 10 μm. *p < 0.05 and ** < 0.01

To better investigate the function of circCRKL, a CML mouse model was created. Sh-NC or sh-circCRKL lentivirus-infected K562/G01 cells were delivered intravenously (i.v.) into immunodeficient NOD/SCID mice, respectively. Prior studies have noted that the features of CML mice containing increased white blood cell count and histopathological variation such as bone marrow, liver, and spleen infiltration caused by leukemic cells [32]. As expected, the white blood cell count of the control group mice increased more than that of the knockdown group, but no distinct difference exists (*p* = 0.0586) (Fig. 3A). In addition, compared with sh-NC group mice, knockdown of circCRKL ameliorated the splenomegaly. However, there was no significant defect for weights of livers in the sh-circCRKL group compared to the NC group (Fig. 3B, C). To evaluate the infiltration of leukemic cells in more depth, we set out to perform Wright’s staining in murine bone marrow, liver, and spleen. The results showed severer infiltration in the sh-NC group xenograft models (Fig. 3D), similarly confirmed by hematoxylin/eosin (HE) staining (Fig. 3E). The BCR-ABL level in mouse BM, liver, and spleen was then assessed with immunofluorescence, and the results revealed a much lower level in circCRKL silencing mice (Fig. 3F). Overall, these findings indicated that circCRKL can promote the malignant progression of CML in vivo.

### circCRKL regulates BCR-ABL expression and miR-877-5p is a target for both circCRKL and BCR-ABL

**Fig. 4:**
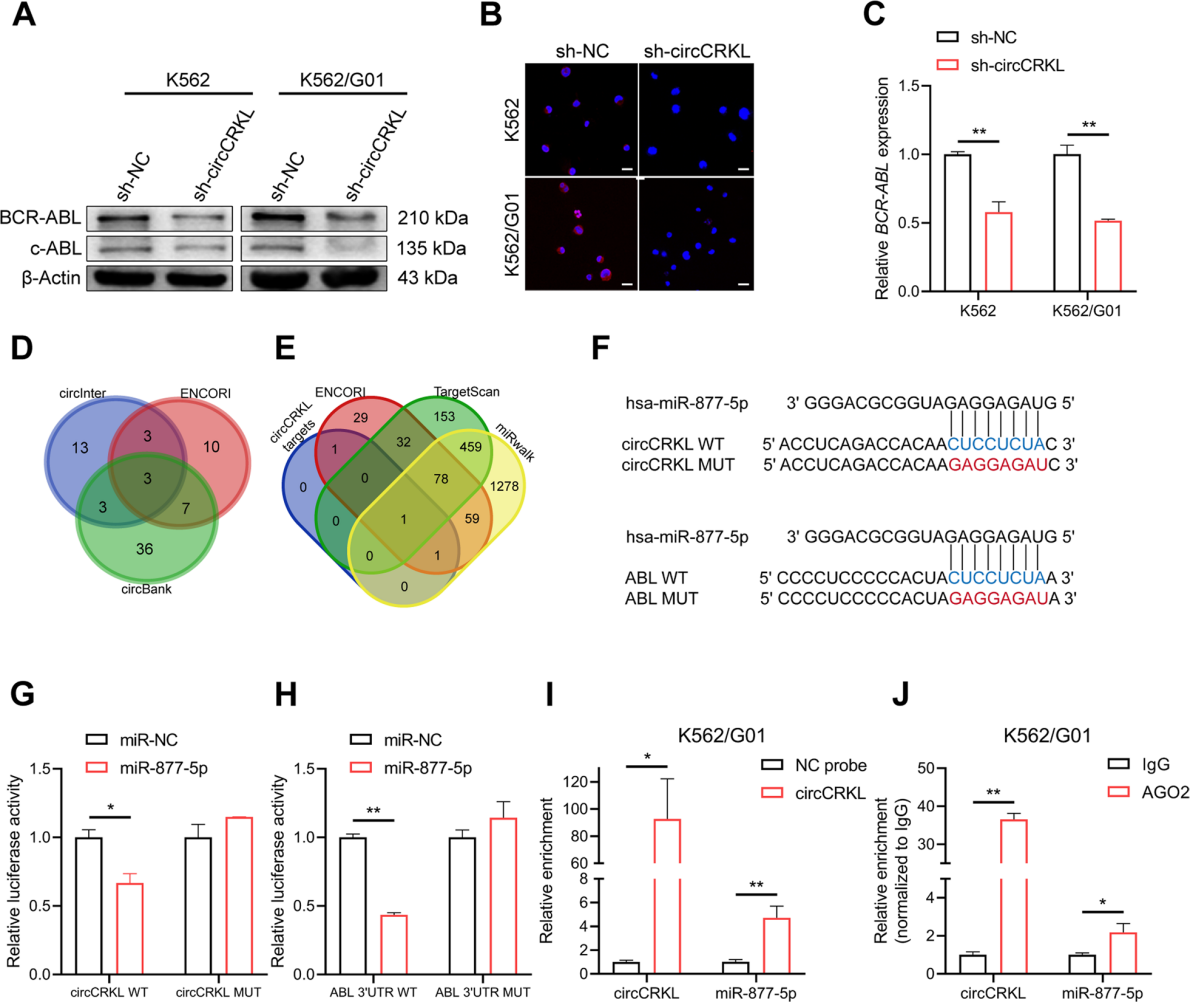
BCR-ABL levels were downregulated in circCRKL silencing CML cells. Effect of circCRKL silencing on BCR-ABL protein levels was evaluated by western bolt assays (**A**) and immunofluorescence (**B**). Scale bar, 10 μm. **C** Effect of circCRKL knockdown on BCR-ABL mRNA levels in CML cells was determined with RT-qPCR. **D** The Venn diagram illustrating the overlapped miRNAs for circCRKL predicted using ENCORI, circInteractome, and circBank. **E** The Venn diagram showed the overlapped miRNAs between circCRKL targets and candidates for ABL obtained from ENCORI, TargetScan, and miRwalk. **F** The schematic diagrams of binding sites of circCRKL with miR-877-5p, and ABL 3’UTR with miR-877-5p. **G**,** H** Relative luciferase activities of the reporter plasmids were assessed in 293 T cells treated with miR-877-5p mimic or miR-NC. **I** Relative enrichment of circCRKL and miR-877-5p on circCRKL probe in K562/G01 cells was evaluated with RNA pulldown assay. **J** Relative enrichment of circCRKL and miR-877-5p on AGO2 protein in K562/G01 cells was detected by RNA immunoprecipitation. *p < 0.05 and ** < 0.01

Since we discovered that circCRKL is essential in BCR-ABL^+^ cells, it’s critical to figure out how circCRKL and BCR-ABL are linked. Notably, western blot showed a reduction of the BCR-ABL and c-ABL protein levels in circCRKL suppressed cells (Fig. 4A). The BCR-ABL expression level was further examined using an immunofluorescence test, and the results were in concert with those previously mentioned (Fig. 4B). Furthermore, RT-qPCR demonstrated that knocking down circCRKL reduced *BCR-ABL* mRNA expression (Fig. 4C), suggesting circCRKL may regulate BCR-ABL at the mRNA level. The mechanism by which circCRKL controls BCR-ABL, however, is yet unknown.

Given that circCRKL is predominantly found in the cytoplasm of CML cells, which is a critical prerequisite for circCRKL to function as a miRNA sponge. Therefore, we screened downstream miRNAs of circCRKL from three databases (circInteractome, circBank, and ENCORI) (Fig. 4D). Subsequently, ENCORI and the other two databases TargetScan and miRwalk were applied to predict miRNAs bound to ABL. Then we overlapped miRNAs of circCRKL and ABL (Fig. 4E). Among candidate miRNAs, miR-877-5p is a unique miRNA containing binding sites for both circCRKL and ABL 3’UTR (Fig. 4F), as confirmed through dual-luciferase reporter assays. The wild-type circCRKL reporter’s luciferase activity was drastically diminished when miR-877-5p was ectopically expressed, however, the mutant reporter’s activity was unaffected, demonstrating that circCRKL may interact with miR-877-5p at specific sites (Fig. 4G), and similar results were noticed between miR-877-5p and ABL (Fig. 4H). The connection between circCRKL and miR-877-5p was further confirmed by RNA pull-down assays. The efficiency of the circCRKL probe was testified by RT-qPCR, then as expected, miR-877-5p was found to be specifically enriched by the circCRKL probe in K562/G01 cells (Fig. 4I). Moreover, prior studies have noted the importance of AGO2 for circRNA to act as a miRNA sponge [33]. Herein, an anti-AGO2 RNA immunoprecipitation was performed, showing both circCRKL and miR-877-5p could interact with AGO2, implying that circCRKL might act as a sponge for miR-877-5p (Fig. 4J).

### miR-877-5p restrains CML cells proliferation via regulating BCR-ABL

**Fig. 5:**
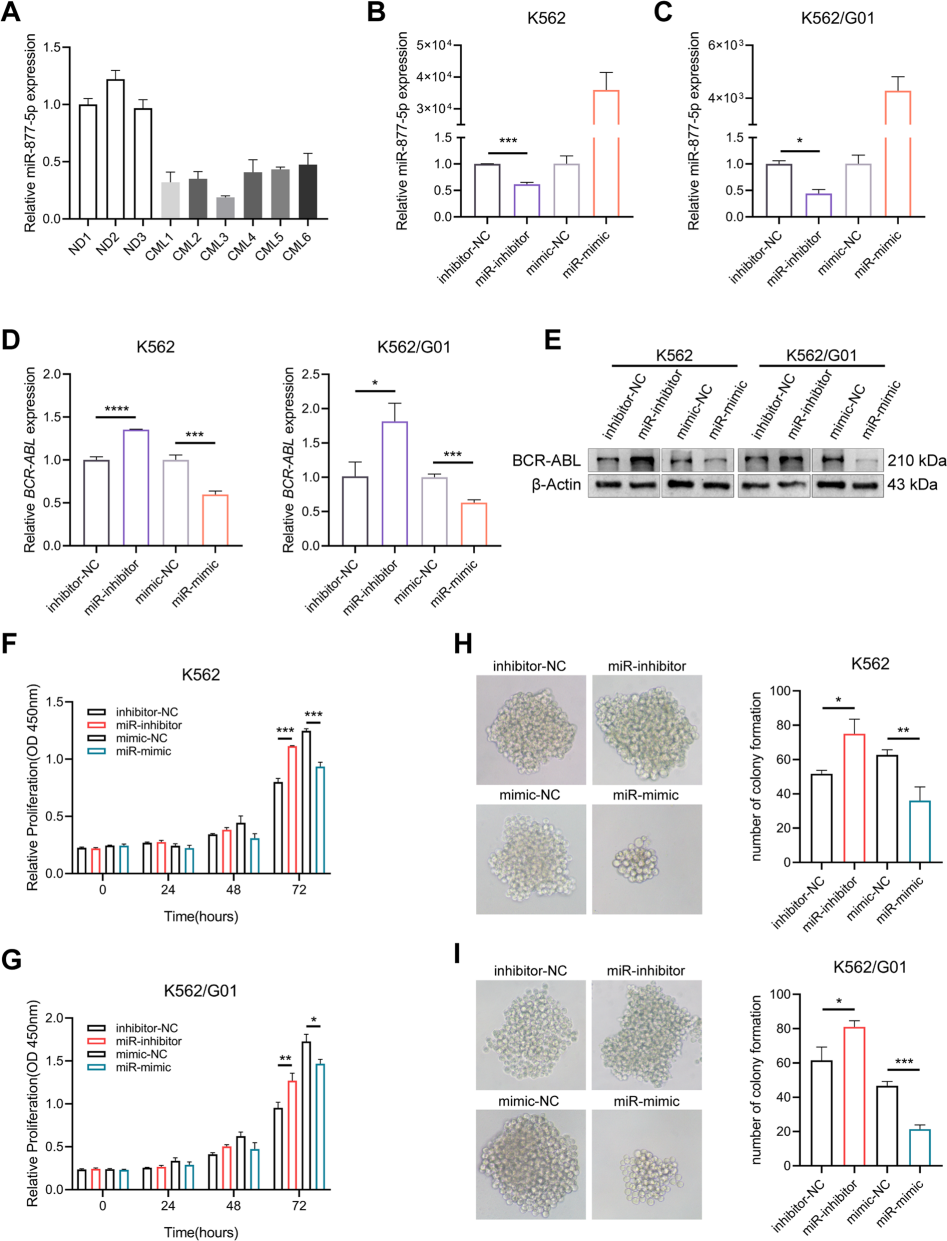
miR-877-5p regulates the BCR-ABL expression. **A** Differential expression of miR-877-5p between CML and normal donors bone marrow samples. The efficiency of miR-877-5p inhibitor or mimic in K562 (**B**) and K562/G01 (**C**) cells were determined with RT-qPCR. Relative *BCR-ABL* mRNA (**D**) and protein (**E**) expression levels were detected by RT-qPCR with transfecting miR-inhibitor or miR-mimic, respectively. **F**,** G** Effect of miR-inhibitor or miR-mimic on viability in CML cells was measured with CCK-8 assays. **H**,** I** Colony formation assays were used for evaluating the effect of miR-inhibitor or miR-mimic on proliferation in CML cells. *p < 0.05, **p < 0.01, ***p < 0.001 and **** < 0.0001

To figure out the biological relevance of miR-877-5p in CML and whether BCR-ABL is controlled by miR-877-5p, the expression level in BMMCs from CML patients was determined (Fig. 5A), then inhibitor or mimics was utilized to knock down or overexpress miR-877-5p, respectively. The effect of inhibitor or mimics on miR-877-5p in CML cells was examined with RT-qPCR (Fig. 5B, C). The *BCR-ABL* mRNA expression was significantly up-regulated by inhibiting miR-877-5p, whereas overexpressing miR-877-5p markedly decreased *BCR-ABL* (Fig. 5D). Likewise, western blot demonstrated that miR-877-5p inhibition augmented BCR-ABL protein level, while miR-877-5p overexpression showed an opposite effect (Fig. 5E). Furthermore, CCK-8 (Fig. 5F, G) and colony formation assays (Fig. 5H, I) revealed that miR-877-5p inhibition stimulated CML cells proliferation and miR-877-5p overexpression had an opposite effect. Altogether, these findings revealed miR-877-5p acts as a negative regulator of BCR-ABL.

### circCRKL promotes CML cells proliferation via sponging miR-877-5p to modulate BCR-ABL

**Fig. 6:**
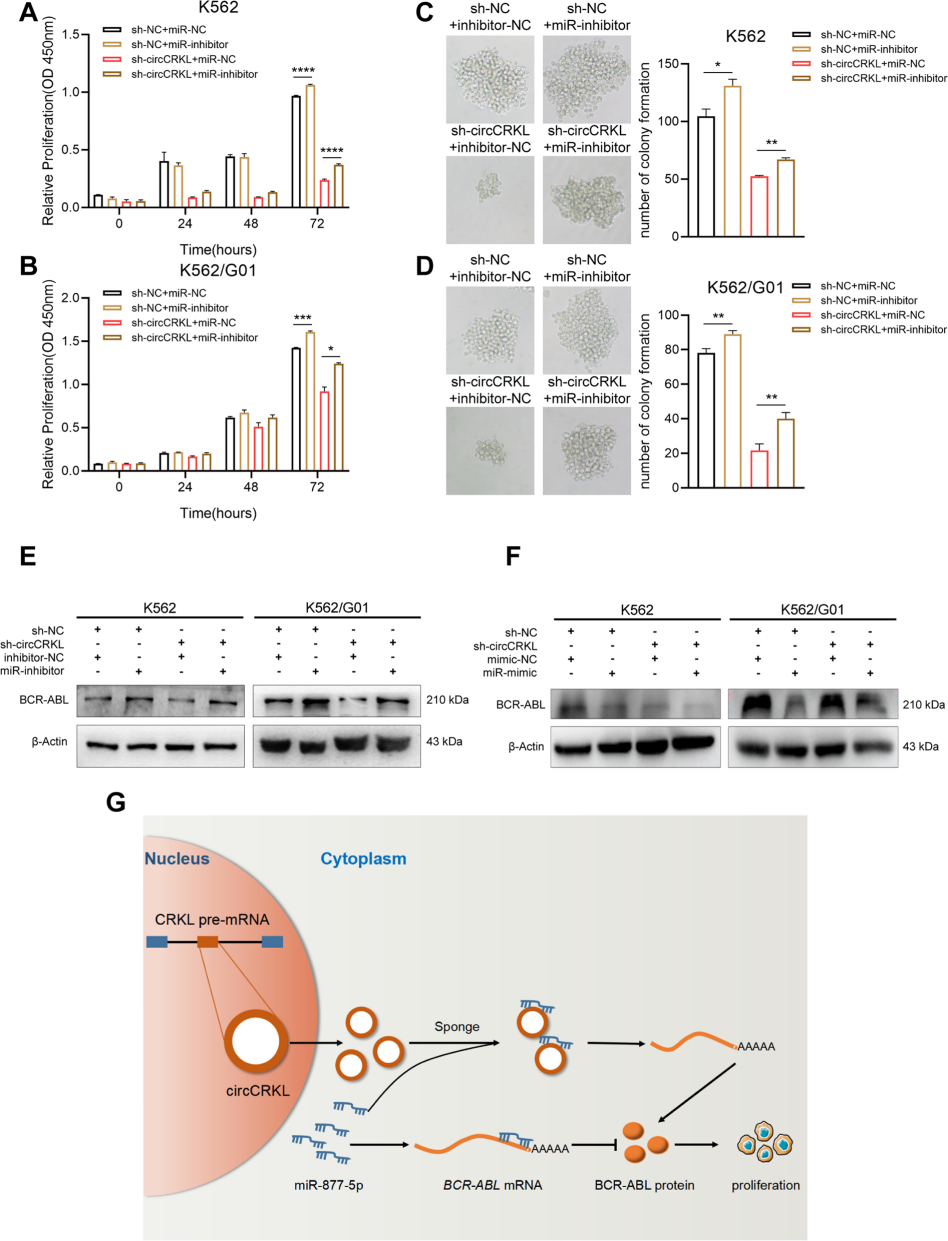
circCRKL absorbs miR-877-5p to augment BCR-ABL and to accelerate CML cells proliferation. **A**,** B** CCK-8 assay was conducted to measure the cell growth co-transfected with circCRKL knockdown lentivirus and miR-877-5p inhibitor. **C**,** D** Colony formation assay was performed to determine the proliferation in CML cells co-transfected circCRKL knockdown lentivirus and miR-877-5p inhibitor. **E** The BCR-ABL protein levels were detected by western blot assays with co-transfecting circCRKL knockdown lentivirus and miR-877-5p inhibitor. **F** The BCR-ABL protein levels were determined using western blot assays with co-transfecting circCRKL knockdown lentivirus and miR-877-5p mimic. **G** Schematic diagram illustrates that circCRKL promotes CML cells proliferation via miR-877-5p/BCR-ABL axis. *p < 0.05, **p < 0.01 and *** < 0.001

To further explore whether circCRKL supports CML cells proliferation by controlling miR-877-5p/BCR-ABL axis. A rescue experiment was performed by co-transfecting sh-circCRKL along with miR-877-5p inhibitor. Knockdown of miR-877-5p distinctly restored the restrained growth induced by suppressing circCRKL in CML cells, according to CCK-8 and colony formation assays (Fig. 6A–D). Moreover, miR-877-5p knockdown markedly ameliorated the reduced BCR-ABL level induced by circCRKL suppression (Fig. 6E). In addition, the function of sh-circCRKL in miR-mimic cells was determined by measuring the BCR-ABL protein level in CML cells coupled with circCRKL knockdown. According to the findings, BCR-ABL protein levels were lower in CML cells co-transfected sh-circCRKL along with miR-mimic than that in sh-circCRKL + mimic-NC cells (Fig. 6F). Collectively, these results proved that circCRKL promotes CML cell proliferation through regulating miR-877-5p mediated BCR-ABL.

### K562/G01 cells are more sensitive to imatinib when circCRKL is knocked down

Given that the imatinib-resistant cell line K562/G01 had higher levels of circCRKL expression than K562 cells. We speculate that a link may exist between circCRKL and imatinib resistance. The half-maximal inhibitory concentration value (IC-50) of K562 or K562/G01 cells against imatinib was measured using a CCK-8 assay (Additional file 2: Figure S2A). Subsequently, the cell viability of stable knockdown of circCRKL and circCRKL wild-type K562/G01 cells treated with imatinib for 48 h was determined and the IC-50 value was computed. Indeed, the data demonstrated that circCRKL silencing observably reduced cell viability, and the IC-50 value was decreased simultaneously (Additional file 2: Figure S2B). In addition, flow cytometry demonstrated that knocking down circCRKL boosted the apoptosis in K562/G01 cells treated with imatinib (Additional file 2: Figure S2C). We assumed that miR-877-5p might be implicated in imatinib-resistance, hence the impact of suppressing or overexpressing miR-877-5p on the IC-50 value of K562/G01 cells was then investigated. However, no distinct effect on IC-50 value was caught (Additional file 2: Figure S2D). Previous investigations have linked the Wnt pathway with imatinib-resistance [34, 35], the relevant markers including CTNNB1 and c-Myc were detected using a western blot assay, accordingly. The data demonstrated a discernible loss of both CTNNB1 and c-Myc in K562/G01 cells which circCRKL was knocked down (Additional file 2: Figure S2E). Collectively, these findings indicated that circCRKL may regulate the susceptibility of K562/G01 cells to imatinib through mediating the Wnt/β-catenin signaling pathway.

## Discussion

*BCR-ABL* is a fusion gene that leads to the occurrence and progression of CML [2, 36]. Despite that tyrosine kinase inhibitors such as imatinib targeting BCR-ABL oncoprotein have ameliorated survival effectively [6], the TKI-resistance and uncontrolled progression prevent part of CML patients from curative benefit [8, 37]. Therefore, novel treatment strategies are supposed to be provided for optimizing the outcomes of CML patients.

During the past decade, the evolution of high-throughput sequencing has brought circRNAs to the forefront. circRNAs have been widely developed for therapeutic targets in diverse diseases due to their stable circular structure. An artificial circRNA scRNA21 was applied to sponge oncogenic miR-21 to restrain gastric cancer cell proliferation [38]. More recently, circRNA has been developed into vaccines for the treatment of SARS-CoV-2 [39]. Accumulating evidence has proved that circRNAs generated by cancer-associated parental genes play a vital role in those cancers [40]. For example, it has conclusively been shown that circMYBL2, derived from AML-related gene *MYBL2*, exerts a cancerogenic role in leukemia harboring *FLT3*-ITD mutation [20]. However, few species of investigations on the function of such circRNAs in CML. In this current research, we firstly reported that circCRKL, back spliced from the second exon of pre-*CRKL*, was highly expressed in BMMCs of CML patients and BCR-ABL^+^ cell lines. Previous studies revealed that circCRKL was downregulated and mitigated carcinogenesis in prostate cancer and acute myeloid leukemia [29, 30]. The reason circCRKL differential expressed in CML and other cancers could be explained is that circRNAs are cancer-specific and cell type-specific [41]. We hypothesized that circCRKL can function in CML in concert with its parental gene *CRKL*. As expected, further loss of function assays showed that knockdown of circCRKL dampened CML cells proliferation in cell lines and mouse models, suggesting circCRKL exerts an oncogenic role in CML.

circRNAs located in the cytoplasm can absorb specific miRNAs to control the related target genes, and to mediated tumorigenesis and progression. For example, circCDYL sponges miR-145-5p to augment tumor-suppressor TJP1 in Wilm’s tumor [42]. circRNF220 was identified as a key upstream regulator of miR-30a, leading to elevated expression of MYSM1 and IER2 in AML cells [43]. In this study, we noted that circCRKL is predominantly expressed in the cytoplasm of CML cell lines. Intriguingly, we found that knockdown of circCRKL markedly diminished BCR-ABL in both mRNA and protein levels. Given that circCRKL could function as a miRNAs sponge [21], the hypothesis was established that circCRKL regulates BCR-ABL expression level via specific miRNA. Hence, several bioinformatics databases were utilized to predict the target miRNAs of circCRKL and BCR-ABL simultaneously. Then we found a miRNA, miR-877-5p, containing binding sites of both circCRKL and ABL.

Several studies uncovered that miR-877-5p serves as a suppressor in a diversity of cancers. Yu et al. discovered that circ_0061395 promoted HCC advancement by sponging miR-877-5p and increasing PIK3R3 expression [44]. Luo et al. uncovered that miR-877-5p restrains PDAC progression via negatively regulating lncRNA NR2F1-AS1 [45]. Additionally, researchers have confirmed miR-877-5p as a positive element in other malignancies [46, 47]. Herein, we firstly reported the involvement of miR-877-5p in CML. The RNA pull-down and dual-luciferase reporter assays illustrate that circCRKL exerts a sponge effect on miR-877-5p. Furthermore, we discovered that in CML, miR-877-5p was downregulated, and miR-877-5p silencing could offset circCRKL knockdown-mediated the proliferation restrain in CML cells. More than that, deep investigations demonstrated that miR-877-5p silencing elevated BCR-ABL expression level, and reverse results were induced by ectopic expressing miR-877-5p. Additionally, rescue experiments demonstrated that inhibition of miR-877-5p ameliorates the reduced BCR-ABL caused by knockdown of circCRKL, which validated the hypothesis we raised. Collectively, we revealed that circCRKL could sponge miR-877-5p to regulate BCR-ABL expression level to maintain the malignancy of CML cells. However, the present research is predominantly at the stage of cell and animal trials, additional studies were required to determine whether the target can be applied to CML individuals.

## Conclusions

In summary, our studies uncover circCRKL, generated from CML-related gene *CRKL*, maintains BCR-ABL expression level by sponging miR-877-5p, thus facilitating CML cell proliferation (Fig. 6G). Besides, we reveal that circCRKL plays a crucial role in imatinib-resistant cells (Additional file 2: Figure S2), which provides a novel perspective for easing the drug resistance of CML patients. However, extensive investigation to uncover the molecular basis of circCRKL involved in imatinib resistance should be undertaken. Overall, our work suggests that circCRKL can lead the progression of a therapeutic target for CML.

## Supplementary Information

**Additional file 1:** **Fig. S1. **circCRKL is required in BCR-ABL^+^ cells.** A. **The BCR-ABL levels in various cell lines were determined with western blot assays. **B. **Effect of circCRKL suppression on apoptosis in CML cells was assessed by flow cytometry. **C. **RT-qPCR was performed to detect the efficiency of circCRKL knockdown in KCL22, SupB15, THP-1, and TK-6 cell lines. **D-G. **CCK-8 assays were used to assess the effect of circCRKL suppression on cell viability in KCL22 **(D)**, SupB15 **(E)**, THP-1 **(F)**, and TK-6 **(G) **cells. *p < 0.05, **p < 0.01 and *** < 0.001.

**Additional file 2:** **Fig. S2. **circCRKL knockdown ameliorates the sensitivity of imatinib-resistant cell line K562/G01.** A. **The cell survival rate and IC50 value were calculated with a CCK-8 assay after treatment with imatinib for 48 hours at different concentrations in K562 and K562/G01 cells. **B.** The cell survival rate and IC50 value were calculated with a CCK-8 assay after treatment with imatinib for 48 hours at different concentrations in K562/G01 cells knocked down circCRKL or its normal control. **C. **The apoptosis rate was measured with flow cytometry 48 hours after treatment with imatinib at 5 μM concentration. **D. **The cell survival rate and IC50 value were calculated with a CCK-8 assay after treatment with imatinib for 48 hours at different concentrations in K562/G01 cells suppressed or overexpressed miR-877-5p. **E.** Relative protein levels were determined with western blot assays. *p < 0.05 and ** < 0.01.

**Additional file 3: Table S1. **Clinical patients information.** Table S2. **Primers sequences used in RT-qPCR assay. **Table S3. **The siRNA sequences used for knocking down circCRKL. **Table S4. **The probe sequences used for RNA pull-down assay.

## Data Availability

Not applicable.

## Abbreviations

CML: Chronic myeloid leukemia
circRNA: Circular RNA
BCR-ABL^+^: BCR-ABL positive
BCR-ABL^−^: BCR-ABL negative
miRNA: Micro RNA
TKI: Tyrosine kinase inhibitor
IM: Imatinib
IC50: Half-maximal inhibitory concentration
RPMI1640: Roswell Park Memorial Institute-1640
DMEM: Dulbecco's modified eagle medium
FBS: Fetal bovine serum
BMMCs: Bone marrow mononuclear cells
FISH: Fluorescence in situ hybridization
RIP: RNA Immunoprecipitation
