# Supplementary material for: circCRKL, a circRNA derived from CRKL, regulates BCR-ABL via sponging miR-877-5p to promote chronic myeloid leukemia cell proliferation
Source: J Transl Med. 2022 Sep 4;20:395. doi: 10.1186/s12967-022-03586-2 (PMC9440867; doi:10.1186/s12967-022-03586-2)
Supplement: Supplementary file 1 — Additional file 1: Fig. S1. circCRKL is required in BCR-ABL+ cells. A. The BCR-ABL levels in various cell lines were determined with western blot assays. B. Effect of circCRKL suppression on apoptosis in CML cells was assessed by flow cytometry. C. RT-qPCR was performed to detect the efficiency of circCRKL knockdown in KCL22, SupB15, THP-1, and TK-6 cell lines. D-G. CCK-8 assays were used to assess the effect of circCRKL suppression on cell viability in KCL22 (D), SupB15 (E), THP-1 (F), and TK-6 (G) cells. *p < 0.05, **p < 0.01 and *** < 0.001. [file 12967_2022_3586_MOESM1_ESM.docx]

**Figure. S1**

**
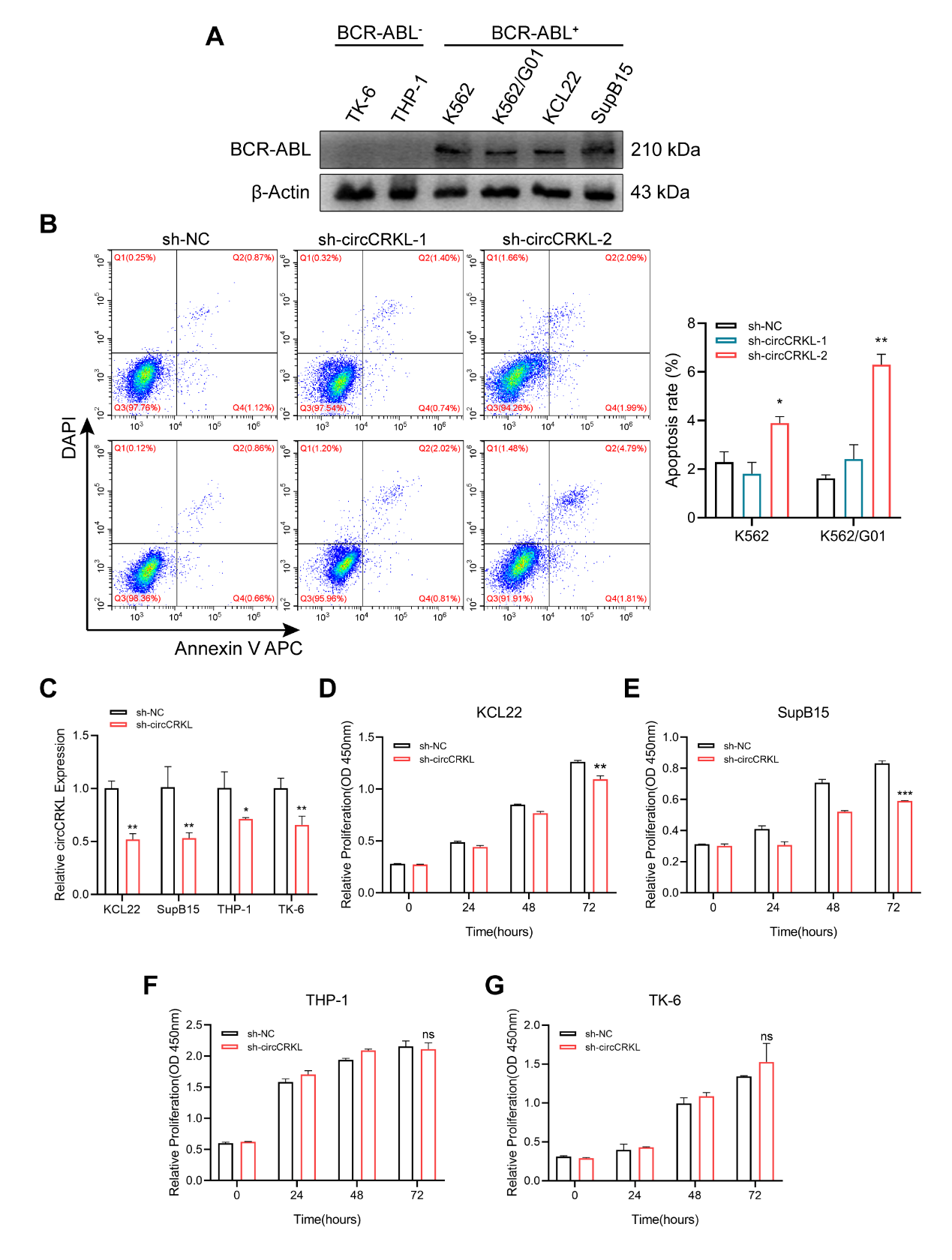
**

**Fig. S1 circCRKL is required in BCR-ABL^+^ cells. A.** The BCR-ABL levels in various cell lines were determined with western blot assays. **B.** Effect of circCRKL suppression on apoptosis in CML cells was assessed by flow cytometry. **C.** RT-qPCR was performed to detect the efficiency of circCRKL knockdown in KCL22, SupB15, THP-1, and TK-6 cell lines. **D-G.** CCK-8 assays were used to assess the effect of circCRKL suppression on cell viability in KCL22 **(D)**, SupB15 **(E)**, THP-1 **(F)**, and TK-6 **(G)** cells. **p* < 0.05, ***p* < 0.01 and *** < 0.001.
