# Supplementary material for: circCRKL, a circRNA derived from CRKL, regulates BCR-ABL via sponging miR-877-5p to promote chronic myeloid leukemia cell proliferation
Source: J Transl Med. 2022 Sep 4;20:395. doi: 10.1186/s12967-022-03586-2 (PMC9440867; doi:10.1186/s12967-022-03586-2)
Supplement: Supplementary file 2 — Additional file 2: Fig. S2. circCRKL knockdown ameliorates the sensitivity of imatinib-resistant cell line K562/G01. A. The cell survival rate and IC50 value were calculated with a CCK-8 assay after treatment with imatinib for 48 hours at different concentrations in K562 and K562/G01 cells. B. The cell survival rate and IC50 value were calculated with a CCK-8 assay after treatment with imatinib for 48 hours at different concentrations in K562/G01 cells knocked down circCRKL or its normal control. C. The apoptosis rate was measured with flow cytometry 48 hours after treatment with imatinib at 5 μM concentration. D. The cell survival rate and IC50 value were calculated with a CCK-8 assay after treatment with imatinib for 48 hours at different concentrations in K562/G01 cells suppressed or overexpressed miR-877-5p. E. Relative protein levels were determined with western blot assays. *p < 0.05 and ** < 0.01. [file 12967_2022_3586_MOESM2_ESM.docx]

**Figure. S2**

**
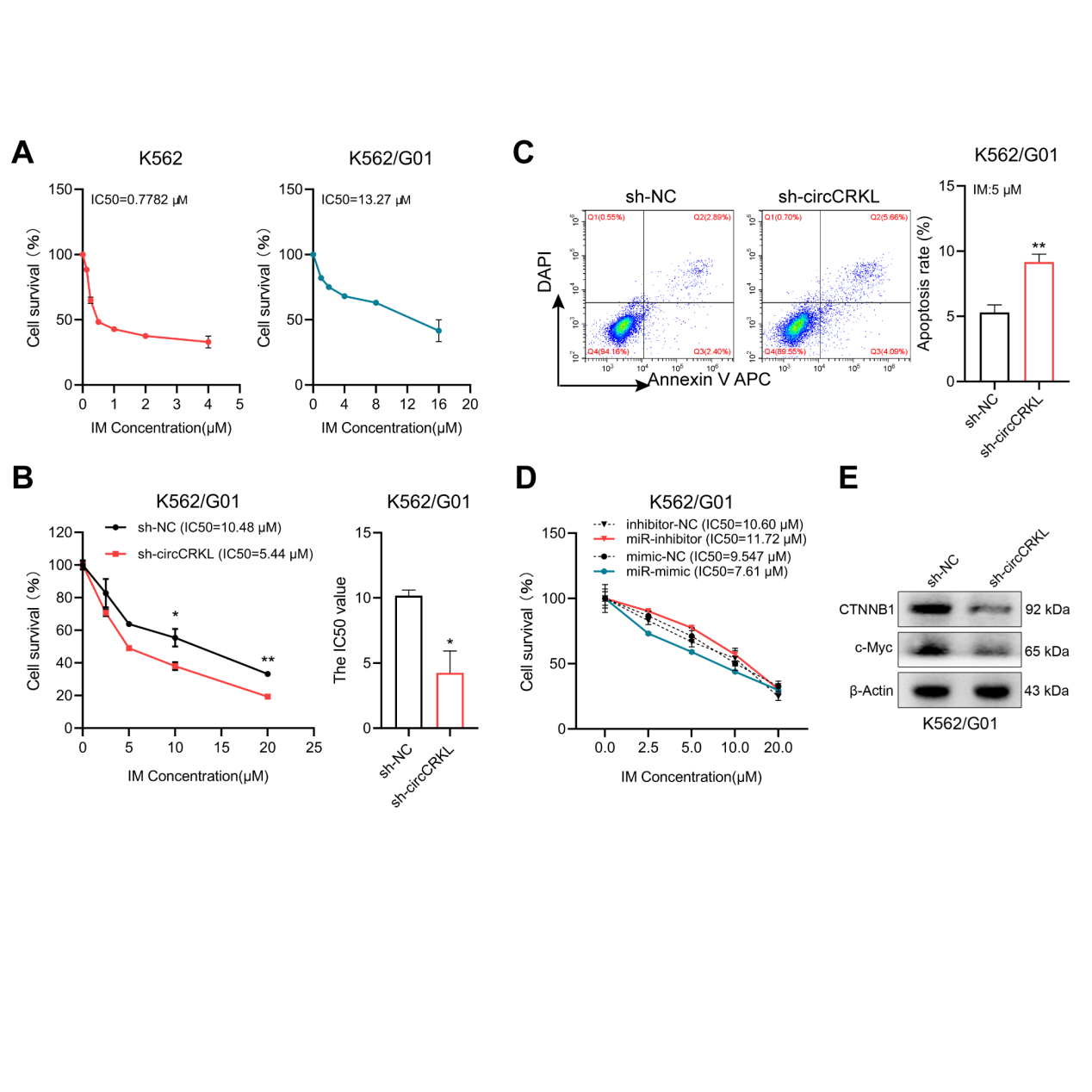
**

**Fig. S2 circCRKL knockdown ameliorates the sensitivity of imatinib-resistant cell line K562/G01. A.** The cell survival rate and IC50 value were calculated with a CCK-8 assay after treatment with imatinib for 48 hours at different concentrations in K562 and K562/G01 cells. **B.** The cell survival rate and IC50 value were calculated with a CCK-8 assay after treatment with imatinib for 48 hours at different concentrations in K562/G01 cells knocked down circCRKL or its normal control. **C.** The apoptosis rate was measured with flow cytometry 48 hours after treatment with imatinib at 5 μM concentration. **D.** The cell survival rate and IC50 value were calculated with a CCK-8 assay after treatment with imatinib for 48 hours at different concentrations in K562/G01 cells suppressed or overexpressed miR-877-5p. **E.** Relative protein levels were determined with western blot assays. **p* < 0.05 and ** < 0.01.
