# Supplementary material for: circCRKL, a circRNA derived from CRKL, regulates BCR-ABL via sponging miR-877-5p to promote chronic myeloid leukemia cell proliferation
Source: J Transl Med. 2022 Sep 4;20:395. doi: 10.1186/s12967-022-03586-2 (PMC9440867; doi:10.1186/s12967-022-03586-2)
Supplement: Supplementary file 3 — Additional file 3: Table S1. Clinical patients information. Table S2. Primers sequences used in RT-qPCR assay. Table S3. The siRNA sequences used for knocking down circCRKL. Table S4. The probe sequences used for RNA pull-down assay. [file 12967_2022_3586_MOESM3_ESM.docx]

**Table S1. Clinical patients information.**

| **Samples** | **Diagnosis** | **Stage of disease** | **Gender/Age** |
| --- | --- | --- | --- |
| CML1 | Primary | CP | Male/63 |
| CML2 | Primary | CP | Male/44 |
| CML3 | Primary | CP | Male/32 |
| CML4 | Primary | CP | Male/70 |
| CML5 | Relapse | AP | Male/45 |
| CML6 | Relapse | CP | Female/41 |
| Normal1 | Anemia |  | Male/46 |
| Normal2 | Anemia |  | Male/29 |
| Normal3 | Anemia |  | Female/25 |

**Table S2. Primers sequences used in RT-qPCR assay.**

| Gene |  |  |  |  | Sequences |
| --- | --- | --- | --- | --- | --- |
| circCRKL-Foward |  |  |  |  | ACCTGCTCATGCATACGCTC |
| circCRKL-Reverse |  |  |  |  | AGATCCCATTGGTGGGCTTG |
| CRKL-Foward |  |  |  |  | GTATGTTCCTCGTCCGCGAT |
| CRKL-Reverse |  |  |  |  | AGATCCCATTGGTGGGCTTG |
| BCR-ABL-Foward |  |  |  |  | GCTCTATGGGTTTCTGAATGTC |
| BCR-ABL-Reverse |  |  |  |  | TGGCGTGATGTAGTTGCTTG |
| miR-877-5p-Foward |  |  |  |  | CGCGGTAGAGGAGATGGC |
| miR-877-5p-Reverse |  |  |  |  | AGTGCAGGGTCCGAGGTATT |
| Actin-Foward |  |  |  |  | ACTTAGTTGCGTTACACCCTT |
| Actin-Reverse |  |  |  |  | TGTCACCTTCACCGTTCC |
| U6-Foward |  |  |  |  | CTCGCTTCGGCAGCACA |
| U6-Reverse |  |  |  |  | AACGCTTCACGAATTTGCGT |

**Table S3. The siRNA sequences used for knocking down circCRKL**

| Sh-circCRKL-1 | CTTGGCATTAGAGGTATCCAA |
| --- | --- |
| Sh-circCRKL-2 | CATTAGAGGTATCCAAGCCCA |

**Table S4. The probe sequences used for RNA pull-down assay**

| Probe names |  |  |  |  | Sequences |
| --- | --- | --- | --- | --- | --- |
| NC-probe |  |  |  |  | /5bio/-GCGACTTCTAACCGTTAATC |
| circCRKL probe-1 |  |  |  |  | /5bio/-TGGATACCTCTAATGCCAAG |
| circCRKL probe-2 |  |  |  |  | /5bio/-CTTGGATACCTCTAATGCCA |
